# Application of the AT(N) and Other CSF Classification Systems in Behavioral Variant Frontotemporal Dementia

**DOI:** 10.3390/diagnostics13030332

**Published:** 2023-01-17

**Authors:** Vasilios C. Constantinides, Fotini Boufidou, Mara Bourbouli, Efstratios-Stylianos Pyrgelis, Apostolia Ghika, Christos Koros, George Liakakis, Sokratis Papageorgiou, Leonidas Stefanis, George P. Paraskevas, Elisabeth Kapaki

**Affiliations:** 1 First Department of Neurology, School of Medicine, National and Kapodistrian University of Athens, Eginition Hospital, Vass. Sophias Ave. 74, 11528 Athens, Greece; 2 Neurochemistry and Biological Markers Unit, First Department of Neurology, School of Medicine, National and Kapodistrian University of Athens, Eginition Hospital, Vass. Sophias Ave. 74, 11528 Athens, Greece; 3 Second Department of Neurology, School of Medicine, National and Kapodistrian University of Athens, “Attikon” University General Hospital, Rimini 1, 12462 Athens, Greece

**Keywords:** behavioral variant, frontotemporal dementia, frontotemporal lobar degeneration, Alzheimer’s disease, CSF biomarkers, tau protein, amyloid beta, classification system

## Abstract

Background: Patients with a frontotemporal lobar degeneration (FTLD) usually manifest with behavioral variant frontotemporal dementia (bvFTD). Alzheimer’s disease (AD) may also manifest with a predominant behavioral-dysexecutive syndrome, similar to bvFTD. Cerebrospinal fluid (CSF) biomarkers, such as total tau (τ_T_), phosphorylated tau (τ_P-181_) and amyloid beta with 42 amino-acids (Aβ_42_), can predict AD pathology in vivo. The aim of this study was to compare the τ_T_/Aβ_42_ and τ_P-181_/Aβ_42_ ratios, the BIOMARKAPD/ABSI criteria and the AT(N) classification system in a cohort of bvFTD patients. Methods: A total of 105 bvFTD patients (21 possible bvFTD; 20%) with CSF data, examined from 2008 to 2022, were included. Seventy-eight AD patients and 62 control subjects were included. The CSF biomarkers were measured with Innotest (2008–2017 subcohort) and EUROIMMUN (2017–2022 subcohort) ELISAs. Results: Depending on the classification system, 7.6 to 28.6% of bvFTD had an AD biochemical profile. The τ_T_/Aβ_42_ and τ_P-181_/Aβ_42_ ratios classified more patients as AD compared to the BIOMARKAPD/ABSI and AT(N) systems. The patients with possible bvFTD had higher frequencies of AD compared to the probable bvFTD patients. Conclusions: The four classification criteria of CSF AD biomarkers resulted in differences in AD allocation in this bvFTD cohort. A consensus on the optimal classification criteria of CSF AD biomarkers is pivotal.

## 1. Introduction

The behavioral variant of frontotemporal dementia (bvFTD) is characterized by predominant behavioral dysfunction, which includes disinhibition, apathy, loss of empathy and sympathy, perseverative, stereotyped or compulsive/ritualistic behavior, hyperorality and dietary changes [1]. Additionally, from a cognitive standpoint, it manifests with prominent executive deficits with relative sparing of episodic memory and visuospatial function. This clinical presentation typically has an underlying frontotemporal lobar degeneration (FTLD) pathology, predominantly those of the tau (FTLD-tau) or the TDP43 (FTLD-TDP) subtypes [2]. 

Contrary to this behavioral syndrome, Alzheimer’s disease (AD) typically manifests as an amnestic-predominant cognitive syndrome. However, various atypical, non-amnestic phenotypes of AD, particularly in younger patients, have been recognized and included in recent diagnostic criteria. These phenotypes include a behavioral—frontal predominant variant, which is termed “AD dementia with executive dysfunction” in the NIA-AA diagnostic criteria and “frontal variant of AD” in the IWG-2 criteria [3,4]. 

The in vivo recognition of the underlying pathology in patients with bvFTD is pivotal both from a clinical standpoint, to optimize symptomatic treatment, and from a research standpoint, for accurate patient stratification in clinical trials of specific protein-targeting agents. To this end, biomarkers are essential to differentiate between FTLD and AD pathologies.

A decrease in cerebrospinal fluid (CSF) amyloid beta with 42 amino acids (Aβ_42_), combined with an increase in total tau protein (τ_T_) and phosphorylated tau protein in threonine 181 (τ_P-181_) are considered highly diagnostic of an underlying AD pathology, and have, therefore, been included in recent AD diagnostic criteria. Aβ_42_ is a marker of amyloid pathology, τ_P-181_ of tau pathology and τ_T_ a nonspecific marker of neuronal injury/neurodegeneration [5].

Initial CSF biomarker studies focused on the quantitative differences in single biomarker levels between AD and FTLD patients. These studies, in general, pointed towards an elevation of τ_T_ and τ_P-181_, as well as a decrease in Aβ_42_, in AD patients compared to FTLD patients [6,7,8,9,10,11,12,13]. However, due to the considerable overlap between study groups, these CSF biomarkers, when applied individually, provided suboptimal discrimination between AD and FTLD. Moreover, due to the methodological differences among the studies (e.g., in inclusion criteria and CSF measurement methodology), they resulted in conflicting results [6,7,8,9,10,11,12,13]. 

A second approach, in an effort to increase the diagnostic accuracy, was the application of CSF biomarker ratios, such as τ_T_/Aβ_42_ and τ_P-181_/Aβ_42_ [13,14,15,16,17]. These ratios provide improved discrimination between AD and FTLD compared to single CSF biomarkers measurements and were considered optimal biomarkers for this purpose by several CSF/neuropathological studies.

A third approach shifted the conceptual framework of biomarker interpretation from a quantitative-based approach to a qualitative approach, by implementing cut-off points for each biomarker, resulting in a binary (i.e., normal vs. abnormal) classification. This approach has been implemented by the BIOMARKAPD and ABSI recommendations, as well as the AT(N) classification system [18,19]. Both the BIOMARKAPD and the ABSI recommendations require the presence of abnormal values in all three CSF biomarkers (i.e., Aβ_42_, τ_T_ and τ_P-181_) for an AD diagnosis. The AT(N) classification system classifies each biomarker in three groups, namely, amyloid pathology (A), tau pathology (T) and neuronal injury/neurodegeneration (N) [5]. These three biomarker groups can result in eight different biomarker profiles, which can be further classified into three biomarker categories: normal AD biomarkers, Alzheimer’s continuum (which includes Alzheimer’s disease, Alzheimer’s pathologic change and Alzheimer’s and concomitant suspected non-Alzheimer’s pathologic change) and non-AD pathologic change. The AT(N) has some theoretical advantages over other classification systems, since it can provide information on co-pathologies as well as on the temporal evolution of Alzheimer’s disease biomarkers in the preclinical stages.

The introduction of CSF AD biomarkers has significantly improved our understanding of AD both from a pathophysiological and a clinical point of view. However, due to the significant variability in the pre-analytical and analytical factors, as well as the lack of healthy control groups among studies, the CSF biomarker cut-off points vary across different research centers. Moreover, the presence of multiple different criteria for biomarker classification further confounds accurate biomarker interpretation. 

The primary aim of this study was the implementation of four different classification systems of CSF biomarkers (the τ_T_/Aβ_42_ ratio, the τ_P-181_/Aβ_42_ ratio, the BIOMARKAPD/ABSI recommendations and the AT(N) classification system) in a cohort of bvFTD patients examined in a tertiary referral hospital and to present the possible differences among them in AD identification. 

The secondary aims of this study were (a) to compare the frequencies of AD pathology between the possible and probable bvFTD patients and (b) to calculate optimal cut-off points of the CSF biomarkers for the differentiation of bvFTD from AD patients, after exclusion of bvFTD patients with a CSF AD biochemical profile. 

## 2. Materials and Methods

### 2.1. Patients

The medical files of all patients hospitalized from 2008 to 2022 at the “Neurodegenerative Disorders and Epilepsy Ward” of the First Department of Neurology, National and Kapodistrian University of Athens, were screened for cases with a predominant frontal-dysexecutive and/or behavioral syndrome. For these cases, structural (MRI or CT) and functional (HMPAO-Spect) imaging data were also retrieved. These data (clinical and imaging) were retrospectively reviewed individually by four neurologists (V. C., C. K., A. G. and G. L.), and a final diagnosis was set for each patient based on the established diagnostic criteria (i.e., possible bvFTD vs. probable bvFTD vs. other diagnoses) [1]. Based on the established diagnostic criteria, a patient fulfilled the criteria for *possible bvFTD* if he/she exhibited progressive deterioration in behavior and/or cognition and manifested early symptoms in at least three of the following six domains: (a) behavioral inhibition; (b) apathy/inertia; (c) loss of empathy/sympathy; (d) perseverative/stereotyped/compulsive behavior; (e) hyperorality/dietary changes; and (f) neuropsychological deficits in executive tasks with relative sparing of episodic memory and visuospatial skills. A patient fulfilled the criteria for *probable bvFTD* if, in addition to the aforementioned criteria, he/she exhibited a significant functional decline and there were structural or functional imaging studies that supported the diagnosis. Only patients with a consensus on the final diagnosis among the four neurologists were included for the purposes of this study. Of these patients, only the bvFTD patients with available CSF AD biomarkers (τ_T_, τ_P-181_ and Aβ_42_) were included in the analyses. The patients with a positive family history for dementia or motor neuron disease were excluded. 

For comparison, all patients examined from 2008 to 2022 with probable AD dementia with an increased level of certainty (documented decline) and evidence of the AD pathophysiological process, according to the most recent established diagnostic guidelines for AD, were included [3]. The criterion used for evidence of the AD pathophysiological process was the presence of abnormal τ_T_, τ_P-181_ and Aβ_42_ based on the cut-off values of the Neurochemistry and Biomarkers Unit of our department.

Additionally, a control subgroup was included, consisting of otherwise healthy subjects undergoing knee or hip joint surgery or hernia repair under spinal anesthesia. These subjects had no clinical evidence of any major disease and had a negative history of cognitive or behavioral/psychiatric disorder. Moreover, they all had normal scores upon neuropsychological testing (Mini Mental State Examination and Frontal Assessment Battery) prior to operation.

### 2.2. CSF Sampling and Biomarker Measurements

All patients underwent lumbar puncture at 10–11 am, after overnight fasting, according to standard operating procedures in compliance with recommendations to standardize the pre-analytical confounding factors in the AD CSF biomarkers [20]. 

Two cohorts were included in this study. For the initial cohort (patients examined from 2008 to 2017), measurements of Aβ_42_, τ_T_ and τ_P-181_ were performed in duplicate by a double sandwich, enzyme-linked immunosorbent assay (ELISA), as provided by commercially available kits (“Innotest^®^ hTau antigen”, “β- amyloid1–42” and “phospho-tau181” respectively, Innotest Europe, Gent, Belgium), according to the manufacturer’s instructions. These biomarkers were transformed into binary variables (i.e., normal or abnormal), based on cut-off values of the Unit of Neurochemistry and Biomarkers (Aβ_42_ < 682 pg/mL; τ_T_ > 376 pg/mL; τ_P-181_ > 57.76 pg/mL; τ_T_/Aβ_42_ > 0.6628; and τ_P-181_/Aβ_42_ > 0.0904) [21]. 

The second cohort consisted of patients examined from 2017 to 2022. The CSF biomarkers Aβ_42_, τ_T_ and τ_P-181_ were measured in duplicate with ELISA by commercially available kits (EUROIMMUN Beta-Amyloid (1–42) ELISA, EUROIMMUN Total-Tau ELISA and EUROIMMUN pTau (181) ELISA, respectively), according to the manufacturer’s instructions. The CSF biomarkers were transformed into binary variables (i.e., normal or abnormal) based on the cut-off values of the Unit of Neurochemistry and Biomarkers (Aβ_42_ < 480 pg/mL; τ_T_ > 400 pg/mL; τ_P-181_ > 60 pg/mL; τ_T_/Aβ_42_ > 0.710; and τ_P-181_/Aβ_42_ > 0.205) [22]. 

Thirty-five patients had CSF data on both ELISAs (AD: *n* = 21; bvFTD: *n* = 14), resulting in a greater number of CSF samples compared to subjects.

### 2.3. CSF Biomarker-Based Classification Systems

Four different classification criteria were applied based on the CSF biomarkers. Firstly, the AT(N) taxonomic system which, in short, classifies biomarkers of diverse modalities (CSF biomarkers, structural and PET imaging) into three categories: (i) A: biomarkers of amyloid pathology, including Aβ_42_; (ii) T: biomarkers of tau pathology, including τ_P-181_; (iii) (N): biomarkers of neuronal injury/neurodegeneration, including τ_T_. These CSF biomarkers were transformed into binary variables (i.e., normal vs. abnormal), by use of the cut-off values of our lab (see Section 2.2). Three major biomarker categories were produced: (a) “normal biomarkers”: A(−)T(−)N(−); (b) “non-AD pathologic change”: A(−)T(+/−)N(+/−); (c) “Alzheimer’s continuum”: A(+)T(+/−)N(+/−). This last biomarker category was further subdivided into (i) “Alzheimer’s pathologic change”: A(+)T(−)N(−); (ii) “Alzheimer’s disease”: A(+)T(+)N(+/−); (iii) “Alzheimer’s and concomitant suspected non-Alzheimer’s pathologic change”: A(+)T(−)N(+).

Secondly, the criteria of the BIOMARKAPD and Alzheimer’s Biomarkers Standardization Initiative (ABSI) recommendations were applied [18,19]. These recommendations require the presence of abnormal values in all three CSF biomarkers (Aβ_42_, τ_T_ and τ_P-181_) for an AD diagnosis. All other patients were categorized as the “non-AD group”. 

Lastly, the τ_P-181_/Aβ_42_ ratio and the τ_T_/Aβ_42_ ratio criteria were applied. Several studies with antemortem CSF biomarker data and postmortem neuropathological data have supported that these CSF biomarker ratios more accurately reflect the underlying neuropathological lesions and provide the optimal diagnostic accuracy for the differential diagnosis of AD from FTLD [14,23,24]. 

### 2.4. Ethical Considerations

Written informed consent for participation in this study was provided by all patients or their next of kin in cases of compromised mental capacity. This study was approved by the Scientific and Ethics Committee of Eginition Hospital and was performed in accordance with the guidelines of the 1964 Declaration of Helsinki.

### 2.5. Statistical Analysis

Initially, the frequencies of AD vs. non-AD CSF profiles in bvFTD (possible and probable) patients based on the four different classification criteria were calculated. The χ2 test was applied to compare frequencies of AD and non-AD patients among the different classification systems. The comparison among the classification systems included the BIOMARKAPD/ABSI criteria, the τ_T_/Aβ_42_ ratio and the τ_P-181_/Aβ_42_ ratio, since these criteria result in the same classification categories (i.e., AD vs. nonAD). The AT(N) was not included, because it results in multiple CSF biomarker profiles.

The normality of distribution and homogeneity of variances were checked using Shapiro–Wilk’s and Levene’s tests, respectively. The comparison of the clinical, neuropsychological and CSF biomarker characteristics between groups (bvFTD vs. AD vs. controls) was performed by analysis of variance (ANOVA) with Bonferroni correction for multiple analyses or Kruskal–Wallis tests, as appropriate. 

To calculate the optimal cut-off points for the discrimination of bvFTD from AD and of bvFTD from the control subjects, a two-step process was applied, as described elsewhere. Initially, the BIOMARKAPD/ABSI criterion was applied in the bvFTD cohort, and bvFTD patients with an AD CSF biomarker profile were excluded from further analyses. The same criterion was applied for the AD cohort, since only AD patients with an AD CSF biochemical profile according to the BIOMARKAPD/ABSI criterion were included. The BIOMARKAPD/ABSI criterion was selected, because it requires all three CSF biomarkers (Aβ_42_, τ_T_ and τ_P-181_) to be abnormal for an AD diagnosis, thus theoretically providing increased specificity at the expense of sensitivity. 

Secondly, an ROC curve analysis was applied to determine the discriminative power of the CSF biomarkers and biomarker ratios for the differentiation of bvFD vs. AD and bvFTD vs. controls. The cut-off points were determined based on the maximal combined sensitivity and specificity criterion. The area under the curve (AUC), standard deviation of the AUC (SD), 95% confidence interval of the AUC and Youden index were also calculated. For comparison reasons, a stepwise multiple regression analysis was applied in the initial cohort to investigate the contribution of each biomarker (τ_T_, τ_P-181_ and Aβ_42_) in differentiating AD from bvFTD.

All analyses were performed using IBM SPSS Statistics^®^ version 23.0.0.0 (SPSS Inc., Chicago, IL, USA, 2013). All graphs were designed using GraphPad Prism^®^, version 5.03 (GraphPad Software Inc., La Jolla, CA, USA, 2009).

## 3. Results

### 3.1. Clinical and Demographic Data

**Figure 1:**
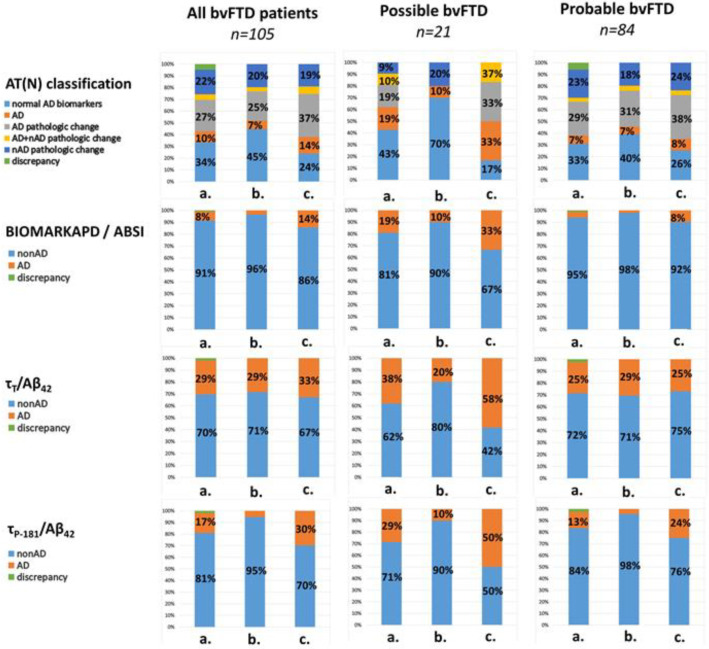
Bar plots of the CSF biomarker profiles in all bvFTD (*n* = 105), possible bvFTD (*n* = 21) and probable bvFTD (*n* = 84) patients. The pies are presented for the whole cohort (a), the 2017–2022 subcohort (b) and the 2008–2017 subcohort (c). Frequencies of <6% are not labeled. nAD: non-Alzheimer’s disease; bvFTD: behavioral variant frontotemporal dementia.

A total of 105 patients with bvFTD were included (45 females; 42.9%). Twenty-one patients (20%) had possible bvFTD and 84 (80%) had probable bvFTD. For comparison, 78 patients with probable AD dementia and 62 control subjects were included (Figure 1, Table 1).

The first cohort (2008–2017; Innotest) consisted of 143 CSF samples (52 probable bvFTD, 12 possible bvFTD, 43 AD and 36 control subjects). The second cohort (2017–2022; Euroimmun) consisted of 138 CSF samples (46 probable bvFTD, 10 possible bvFTD, 56 AD and 26 control subjects). 

The subgroups differed in their age (*p* = 0.004), with the post hoc analysis revealing that the control subjects were older than the probable bvFTD patients (*p* = 0.002). The disease duration did not differ among the study groups. The MMSE scores differed significantly among groups, with AD patients exhibiting the lowest MMSE scores, as expected. The possible bvFTD group had a greater male/female ratio compared to the control subjects and AD (Table 1). 

### 3.2. Comparison of CSF Biomarker Classification Systems

In the total cohort of bvFTD patients (*n* = 105), 9.5% had Alzheimer’s disease and 26.7% had Alzheimer’s pathologic change, according to the AT(N) system. The application of the BIOMARKAPD/ABSI, τ_Τ_/Aβ_42_ and τ_P-181_/Aβ_42_ criteria resulted in 7.6%, 28.6% and 17.1% of bvFTD patients being classified as having a CSF AD biochemical profile, respectively. There was no statistically significant difference between the different classification systems (Figure 1). 

### 3.3. Comparison of Possible and Probable bvFTD Patients

The patients with possible bvFTD had higher frequencies of a CSF AD biochemical profile compared to the probable bvFTD patients. More specifically, 19% of the possible bvFTD patients had an AD profile according to both the AT(N) and BIOMARKAPD/ABSI classification systems. The τ_T_/Aβ_42_ and τ_P-181_/Aβ_42_ ratios resulted in numerically higher CSF AD biochemical profiles among the possible bvFTD patients (38.1% and 28.6%, respectively). The respective frequencies were 7.2%, 4.8%, 25.3% and 12.3% for the probable bvFTD cohort. None of these differences were statistically significant (Figure 1).

### 3.4. Comparison of CSF Biomarkers among Study Groups

**Figure 2:**
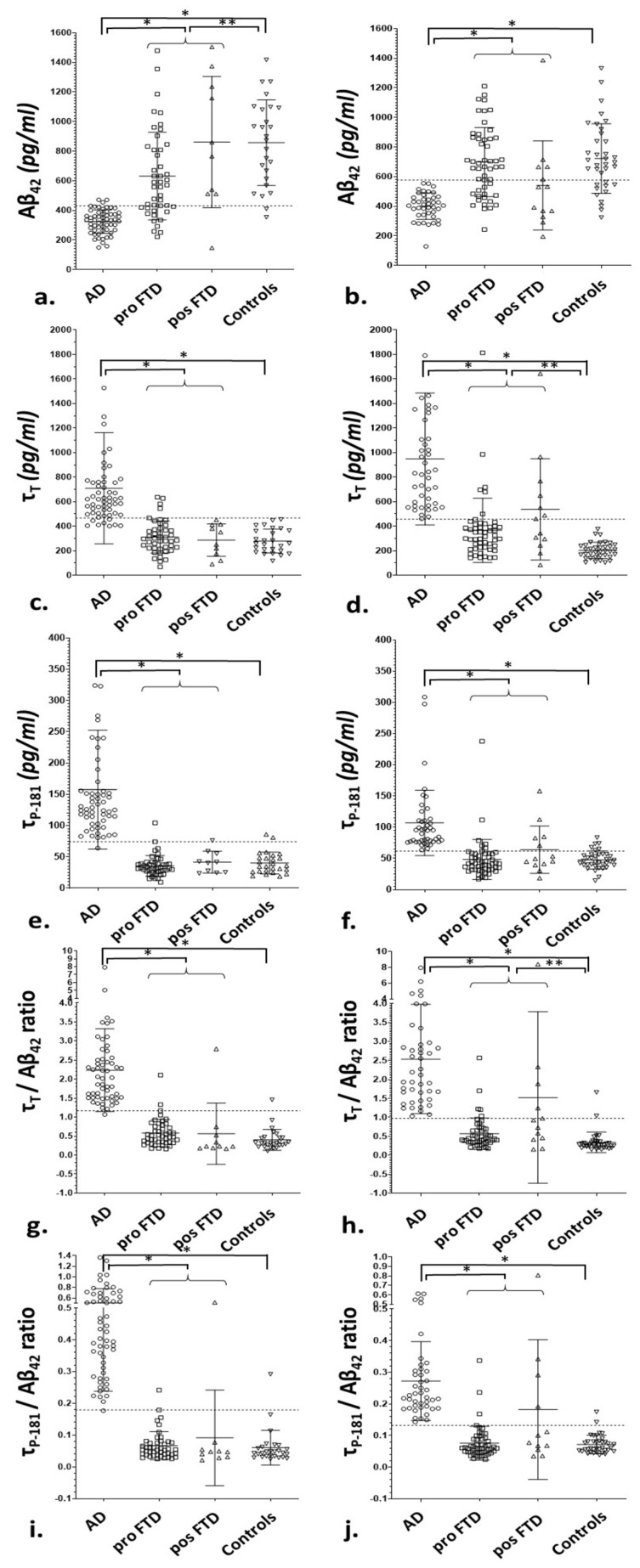
Scatterplots of the CSF biomarkers and their ratios in the 2017–2022 subcohort (**a**,**c**,**e**,**g**,**i**) and the 2008–2017 subcohort (**b**,**d**,**f**,**h**,**j**). The lines and whiskers represent the median values and interquartile range. The dotted lines represent the optimal cut-off points for AD vs. bvFTD discrimination based on the ROC curve analysis. The differences in the post hoc pairwise analyses after Bonferroni correction for multiple comparisons (bvFTD vs. AD; bvFTD vs. controls; AD vs. controls) are included. For the analyses, all bvFTD patients (possible and probable) were used as a single group. For illustrative purposes, the probable bvFTD and possible bvFTD groups are presented separately. * *p* < 0.0001; ** *p* < 0.001.

All CSF biomarkers and biomarker ratios differed significantly among the groups (*p* < 0.001). The post hoc pairwise analyses revealed that AD differed from both the bvFTD and control subjects in all CSF biomarkers and ratios (*p* < 0.0001), both for the Innotest and Euroimmun ELISAs. The bvFTD group differed from the control group in τ_T_ (*p* < 0.001) and τ_T_/Aβ_42_ (*p* < 0.001) using the Innotest and in Aβ_42_ (*p* < 0.001) for the EUROIMMUN ELISA (Table 1, Figure 2).

### 3.5. ROC Curve Analysis

Nine bvFTD patients with an AD biochemical profile, as defined by the BIOMARKAPD/ABSI recommendations, were excluded before the ROC curve analysis. 

**Figure 3:**
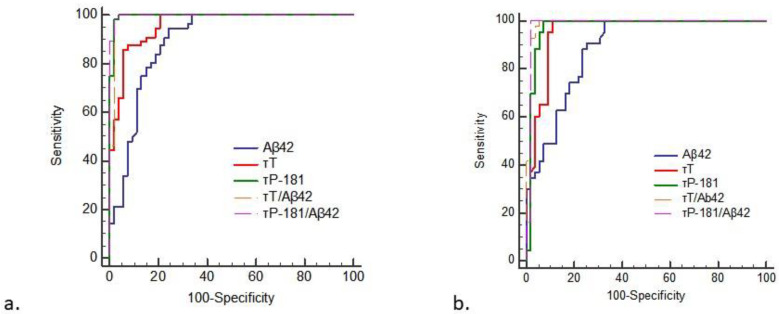
ROC curve analysis of the CSF biomarkers and their ratios for the differentiation of AD from bvFTD. The data are presented for the subcohort of 2017–2022 (**a**) and the subcohort of 2008–2017 (**b**) samples.

For the 2017-2022 cohort, the τ_P-181_/Aβ_42_ ratio provided the optimal discrimination for AD vs. bvFTD (AUC: 0.998; cut-off: >0.1787; sensitivity: 98.2%; specificity: 98.1%). The τ_P-181_ and τ_T_/Aβ_42_ provided comparably high AUCs, sensitivities and specificities (Figure 3 and Table 2).

Stepwise multiple linear regression analysis for the first cohort indicated that AD could be best predicted by the equation

0.559 + (0.004 × τ_P-181_) − (0.001 × Aβ_42_)

The ROC curve analysis of this equation resulted in an AUC of 0.931.

For the 2008–2017 cohort, the τ_T_/Aβ_42_ ratio provided the highest AUC for AD vs. bvFTD discrimination (AUC:0.988; cut-off: >0.976; sensitivity: 100%; specificity: 94.6%). The τ_P-181_ and τ_T_/Aβ_42_ ratios provided comparably high AUCs, sensitivities and specificities (Table 2 and Figure 3).

The stepwise multiple linear regression analysis for this cohort indicated that AD could be best predicted by the equation

0.564 + (0.003 × τ_P-181_) − (0.001 × Aβ_42_)

The ROC curve analysis of this equation resulted in an AUC of 0.985.

## 4. Discussion

In this retrospective, single-center study, we applied four different criteria for CSF biomarker interpretation in a cohort of bvFTD patients. 

An initial finding of our study was that the incidence of AD in the bvFTD cohort varied from 7.6% to 28.6%, depending on the classification criterion applied. Neuropathological studies on the subject have provided comparable results. In the largest cohort of bvFTD patients with a neuropathological confirmation to date, 13% of patients had an AD pathology [2]. In another neuropathological cohort of 86 patients with bvFTD, 17% had AD [25]. However, a smaller clinicopathological study including 32 bvFTD patients reported a single patient (3%) with AD pathology [26]. 

Several studies have focused on CSF biomarker profiles in bvFTD. A study of 43 FTD patients (31 with bvFTD) concluded that 21% had an AD CSF biochemical profile, as defined by a decrease in Aβ_42_ combined with an increase in τ_T_ [27]. Foiani et al. implemented the Aβ_42_ criterion to differentiate between AD and non-AD patients in a cohort of 66 patients with a clinical diagnosis of FTD (21 with bvFTD). Twenty-one patients (31.8%) were classified as AD [28]. However, a study including 14 bvFD patients did not report any patient with an AD CSF profile, based on the τ_T_/Aβ_42_ ratio criterion [29]. 

A neuropathological study of 182 patients focused on the correlations of the AT(N) classification system and the underlying pathology [30]. In this study, none of the 64 autopsy-confirmed FTLD patients had an Alzheimer’s disease profile based on AT(N). Interestingly, only 78% of typical amnestic and 45% of atypical non-amnestic AD patients had an Alzheimer’s disease AT(N) profile. These data imply a high specificity and negative predictive value but a suboptimal sensitivity of the AT(N) criteria in predicting AD pathology. 

A second finding was that the application of the τ_T_/Aβ_42_ and τ_P-181_/Aβ_42_ ratio criteria resulted in higher incidences of AD compared to the AT(N) and BIOMARKAPD/ABSI criteria in our cohort. To the best of our knowledge, studies directly comparing different classification systems in bvFTD cohorts are lacking. We previously applied the same criteria (with the exception of the τ_T_/Aβ_42_ ratio) in a cohort of corticobasal syndrome patients (another atypical phenotype of AD) [22]. In this study, the three classification systems yielded comparable incidences of AD, although the sample size was significantly smaller (*n* = 40). 

In our cohort, the possible bvFTD patients had a greater incidence of AD (19–38.1%) compared to the probable bvFTD patients (4.8–25.3%). This difference may reflect the increased specificity of the probable bvFTD criteria compared to the possible bvFTD criteria, at the expense of sensitivity. This finding was confirmed in a neuropathological study of 117 bvFTD patients [2]. This cohort was classified clinically into low, intermediate and high confidences of a clinical bvFTD diagnosis, with a lower confidence of clinical diagnosis correlated with a higher incidence of AD pathology.

Multiple studies have compared CSF biomarkers quantitatively between AD and bvFTD cohorts in an effort to define the optimal cut-off points for their differentiation. These studies have produced conflicting results, which can be attributed to the differences in the pre-analytical and analytical factors, as well as the inclusion criteria, diagnostic criteria and study designs. Regarding τ_T_, most relevant studies report an increase in bvFTD compared to healthy controls [6,7,11,12,13,31], although some studies have reported no difference [9,10,32]. A single study has reported a decrease in τ_T_ in a subgroup of FTLD patients [8]. Likewise, several studies report a relative decrease in Aβ_42_ levels in FTLD [11,13], whereas others did not find any difference from control groups [10,12,31]. Studies on τ_P-181_ have also provided conflicting results [13,31,32].

A methodological issue in studies investigating possible inherent neurochemical differences between FTLD and AD is the significant clinical–pathological overlap between these two disorders. Thus, particularly for CSF biomarker studies, a two-step approach may provide more robust data on possible CSF biomarker differences. The first step would include the application of a CSF AD classification criterion in order to avoid contamination of typical amnestic patients from non-AD pathologies, as well as of atypical non-amnestic patients from AD. The second step would involve a comparison of quantitative CSF biomarker levels. This two-step approach has been implemented previously, both in CBS cohorts and bvFTD cohorts [33,34].

In order to define the optimal cut-off points for the differentiation of bvFTD from AD, we implemented a two-step algorithm. Initially, we opted to exclude all bvFTD patients with a CSF AD biochemical profile through the use of the BIOMARKAPD/ABSI criterion due to the fact of its increased specificity, which additionally excluded cases with a mixed AD and FTD pathology. We subsequently compared the CSF biomarker levels between this bvFTD cohort and an AD cohort with evidence of the AD pathophysiological process in order to exclude patients with a non-AD underlying pathology and a typical amnestic syndrome.

For the EUROIMMUN assay, the cut-offs values for AD vs. bvFTD discrimination were lower for Aβ_42_ (<430.7 pg/mL vs. <480 pg/mL) and higher for τ_T_ (>467 pg/mL vs. >400 pg/mL) and τ_P-181_ (>74.3 pg/mL vs >60 pg/mL) compared to the established cut-off values of our Neurochemistry and Biomarkers Unit, which are based on AD vs. control subjects differentiation. The Innotest assay resulted in similar results; the cut-off points for AD vs. bvFTD differentiation were lower for Aβ_42_ (<577.1 pg/mL vs. <682 pg/mL) and higher for τ_T_ (>456 pg/mL vs. >376 pg/mL) and τ_P-181_ (>61.9 pg/mL vs. >56.7 pg/mL) compared to the established cut-off values for AD vs. controls differentiation.

By contrast, the bvFTD group differed significantly from the control group in τ_T_ and τ_T_/Aβ_42_ in the 2008–2017 cohort and in Aβ_42_ in the 2017–2022 cohort. However, none of these differences resulted in clinically meaningful differences after the ROC curve analysis. Taken together, these data imply that there may be an inherent mild increase in τ_T_ and τ_P-181_, as well as a mild decrease in Aβ_42_, in bvFTD due to the fact of non-AD pathologies compared to healthy subjects.

A limitation of this study, as is the case with the majority of CSF biomarker studies, is the lack of neuropathological confirmation of diagnoses. To this end, we applied the most recently established diagnostic criteria and relied on a consensus of four neurologists to establish a clinical diagnosis. Moreover, the implementation of a two-step approach, as described previously, assisted in the identification of cases with atypical clinical presentations.

CSF AD biomarkers represent a pivotal step forward, both from a clinical and from a research standpoint. However, difficulties in the application of these biomarkers in everyday clinical practice remain. The standardization of the pre-analytical and analytical factors is the most important step forward in order to achieve reproducible CSF biomarker measurements among different centers and to establish universally accepted cut-off points. Another issue that needs to be addressed is the optimal criteria for CSF biomarker interpretation. To this end, this study presented data on four different classification criteria in a single-center environment. A comparison of these CSF biomarker criteria against neuropathological data would greatly enhance our understanding of the advantages and drawbacks of each of these criteria and their role in clinical practice.

## Figures and Tables

**Table 1:** Clinical, demographic and CSF biomarker data. All data are presented as the mean (SD) or median (25th–75th quartile); *p*-values refer to a comparison between all groups (i.e., AD vs. bvFTD vs. controls); for post-hoc pairwise analysis, *p*-values are included in Figure 2. Pro: probable; Pos: possible. ‡ χ^2^ test; † ANOVA; * Kruskal–Wallis test.

	All bvFTD *n = 105*	Pro bvFTD *n = 84*	Pos bvFTD *n = 21*	AD *n = 78*	Controls *n = 62*	*p*-Value
*Clinical and demographic characteristics*						
Sex *(m/f)*	60/45	43/41	17/4	33/45	24/38	0.005 ‡
Age *(y)*	62.5 (9.8)	62.2 (9.6)	635 (10.7)	649 (10.5)	68.6 (10.4)	0.004 †
Disease duration *(m)*	36 (24–36)	36 (24–54)	30 (24–60)	36 (24–48)	NA	0.984 *
MMSE	19.9 (7.8)	19.2 (7.9)	22.3 (7.2)	18.3 (5.1)	28.7 (1.4)	<0.001 †
*CSF biomarkers*						
*First cohort (2008–2017)*						
	*n = 64*	*n = 52*	*n = 12*	*n = 43*	*n = 36*	
Aβ_42_ *(pg/mL)*	661 (471–852)	667 (495–859)	461 (349–667)	412 (342–477)	689 (545–865)	<0.001 *
τ_T_ *(pg/mL)*	334 (235–429)	321(228–408)	401 (270–600)	826 (577–1189)	195 (153–241)	<0.001 *
τ_P-181_ *(pg/mL)*	43.9 (32.8–57.8)	41.6 (32.0–57.4)	47.5 (40.4–77.8)	94.8 (77.2–110.3)	45.5 (39.4–56.2)	<0.001 *
τ_T_/Aβ_42_	0.47 (0.35–0.79)	0.44 (0.34–0.70)	0.83 (0.44–1.57)	2.12 (1.47–2.92)	0.27 (0.22–0.33)	<0.001 *
τ_P-181_/Aβ_42_	0.065 (0.048–0.100)	0.060 (0.047–0.090)	0.089 (0.061–0.241)	0.2270 (0.189–0.304)	0.060 (0.051–0.081)	<0.001 *
*Second Cohort (2017–2022)*						
	*n = 56*	*n = 46*	*n = 10*	*n = 56*	*n = 26*	<0.001 *
Aβ_42_ *(pg/mL)*	581 (429–854)	565 (421–809)	813 (512–6)	334 (267–380)	886 (613–1093)	<0.001 *
τ_T_ *(pg/mL)*	299 (220–396)	300 (222–384)	301 (176–412)	611 (505–757)	258 (199–364)	<0.001 *
τ_P-181_ *(pg/mL)*	33.2 (26.6–41.9)	32.5 (27.2–41.2)	40.9 (25.3–55.2)	127.5 (105.4–155.3)	35.5 (27.0–48.6)	<0.001 *
τ_Τ_/Aβ_42_	0.47 (0.31–0.75)	0.50 (0.34–0.79)	0.23 (0.19–0.53)	2.03 (1.52–2.52)	0.32 (0.26–0.46)	<0.001 *
τ_P-181_/Aβ_42_	0.053 (0.038–0.077)	0.054 (0.039–0.080)	0.047 (0.031–0.054)	0.429 (0.304–0.650)	0.044 (0.033–0.058)	<0.001 *

**Table 2:** Receiver operating characteristic curve analysis of the CSF biomarkers and their ratios for the differentiation of AD from bvFTD. MLR: multiple linear regression; AUC: area under the curve; CI: confidence interval; Sens: sensitivity; Spec: specificity.

	AUC (SD)	95% CI	*p*-Value	Youden Index	Cut-Off	Sens	Spec
*First cohort (2008–2017)*							
Aβ_42_	0.885 (0.032)	0.804–0.940	<0.001	0.67	<577.1	100	67.3
τ_Τ_	0.950 (0.024)	0.887–0.984	<0.001	0.89	>456.1	100	89.1
τ_P-181_	0.975 (0.018)	0.921–0.996	<0.001	0.93	>61.9	100	92.7
τ_T_/Aβ_42_	0.988 (0.011)	0.941–0.999	<0.001	0.95	>0.976	100	94.6
τ_P-181_/Aβ_42_	0.985 (0.015)	0.936–0.999	<0.001	0.98	>0.1321	100	98.2
MLR equation	0.931 (0.027)	0.865–0.971	<0.001	0.83	>0.34	95.4	87.5
*Second Cohort (2017–2022)*							
Aβ_42_	0.893 (0.033)	0.819–0.944	<0.001	0.70	<430.7	94.6	75.5
τ_T_	0.959 (0.017)	0.903–0.988	<0.001	0.80	>467	85.7	94.3
τ_P-181_	0.995 (0.005)	0.957–1.000	<0001	0.96	>74.3	98.2	98.1
τ_T_/Aβ_42_	0.990 (0.010)	0.948–1.000	<0.001	0.96	>1.169	98.2	98.1
τ_P-181_/Aβ_42_	0.998 (0.0023)	0.962–1.000	<0.001	0.96	>0.1787	98.2	98.1
MLR equation	0.985 (0.01)	0.941–0.999	<0.001	0.91	>0.37	100	91.1

## Data Availability

The data presented in this study are available upon request from the corresponding author.

## References

[1] Rascovsky K., Hodges J.R., Knopman D., Mendez M.F., Kramer J.H., Neuhaus J., van Swieten J.C., Seelaar H., Dopper E.G., Onyike C.U. (2011). Sensitivity of revised diagnostic criteria for the behavioural variant of frontotemporal dementia. Brain.

[2] Perry D.C., Brown J.A., Possin K.L., Datta S., Trujillo A., Radke A., Karydas A., Kornak J., Sias A.C., Rabinovici G.D. (2017). Clinicopathological correlations in behavioural variant frontotemporal dementia. Brain.

[3] McKhann G.M., Knopman D.S., Chertkow H., Hyman B.T., Jack C.R., Kawas C.H., Klunk W.E., Koroshetz W.J., Manly J.J., Mayeux R. (2011). The diagnosis of dementia due to Alzheimer’s disease: Recommendations from the National Institute on Aging-Alzheimer’s Association workgroups on diagnostic guidelines for Alzheimer’s disease. Alzheimer’s Dement..

[4] Dubois B., Feldman H.H., Jacova C., Hampel H., Molinuevo J.L., Blennow K., DeKosky S.T., Gauthier S., Selkoe D., Bateman R. (2014). Advancing research diagnostic criteria for Alzheimer’s disease: The IWG-2 criteria. Lancet Neurol..

[5] Jack C.R., Bennett D.A., Blennow K., Carrillo M.C., Dunn B., Haeberlein S.B., Holtzman D.M., Jagust W., Jessen F., Karlawish J. (2018). NIA-AA Research Framework: Toward a biological definition of Alzheimer’s disease. Alzheimer’s Dement..

[6] Green A.J., Harvey R.J., Thompson E.J., Rossor M.N. (1999). Increased tau in the cerebrospinal fluid of patients with frontotemporal dementia and Alzheimer’s disease. Neurosci. Lett..

[7] Fabre S.F., Forsell C., Viitanen M., Sjogren M., Wallin A., Blennow K., Blomberg M., Andersen C., Wahlund L.O., Lannfelt L. (2001). Clinic-based cases with frontotemporal dementia show increased cerebrospinal fluid tau and high apolipoprotein E epsilon4 frequency, but no tau gene mutations. Exp. Neurol..

[8] Grossman M., Farmer J., Leight S., Work M., Moore P., Van Deerlin V., Pratico D., Clark C.M., Coslett H.B., Chatterjee A. (2005). Cerebrospinal fluid profile in frontotemporal dementia and Alzheimer’s disease. Ann. Neurol..

[9] Bibl M., Mollenhauer B., Wolf S., Esselmann H., Lewczuk P., Kornhuber J., Wiltfang J. (2007). Reduced CSF carboxyterminally truncated Abeta peptides in frontotemporal lobe degenerations. J. Neural. Transm..

[10] Sjogren M., Minthon L., Davidsson P., Granerus A.K., Clarberg A., Vanderstichele H., Vanmechelen E., Wallin A., Blennow K. (2000). CSF levels of tau, beta-amyloid(1-42) and GAP-43 in frontotemporal dementia, other types of dementia and normal aging. J. Neural. Transm..

[11] Riemenschneider M., Wagenpfeil S., Diehl J., Lautenschlager N., Theml T., Heldmann B., Drzezga A., Jahn T., Forstl H., Kurz A. (2002). Tau and Abeta42 protein in CSF of patients with frontotemporal degeneration. Neurology.

[12] Pijnenburg Y.A., Schoonenboom N.S., Rosso S.M., Mulder C., Van Kamp G.J., Van Swieten J.C., Scheltens P. (2004). CSF tau and Abeta42 are not useful in the diagnosis of frontotemporal lobar degeneration. Neurology.

[13] Kapaki E., Paraskevas G.P., Papageorgiou S.G., Bonakis A., Kalfakis N., Zalonis I., Vassilopoulos D. (2008). Diagnostic value of CSF biomarker profile in frontotemporal lobar degeneration. Alzheimer Dis. Assoc. Disord..

[14] Irwin D.J., McMillan C.T., Toledo J.B., Arnold S.E., Shaw L.M., Wang L.S., Van Deerlin V., Lee V.M., Trojanowski J.Q., Grossman M. (2012). Comparison of cerebrospinal fluid levels of tau and Abeta 1-42 in Alzheimer disease and frontotemporal degeneration using 2 analytical platforms. Arch. Neurol..

[15] Rivero-Santana A., Ferreira D., Perestelo-Perez L., Westman E., Wahlund L.O., Sarria A., Serrano-Aguilar P. (2017). Cerebrospinal Fluid Biomarkers for the Differential Diagnosis between Alzheimer’s Disease and Frontotemporal Lobar Degeneration: Systematic Review, HSROC Analysis, and Confounding Factors. J. Alzheimer’s Dis..

[16] Casoli T., Paolini S., Fabbietti P., Fattoretti P., Paciaroni L., Fabi K., Gobbi B., Galeazzi R., Rossi R., Lattanzio F. (2019). Cerebrospinal fluid biomarkers and cognitive status in differential diagnosis of frontotemporal dementia and Alzheimer’s disease. J. Int. Med. Res..

[17] de Souza L.C., Lamari F., Belliard S., Jardel C., Houillier C., De Paz R., Dubois B., Sarazin M. (2011). Cerebrospinal fluid biomarkers in the differential diagnosis of Alzheimer’s disease from other cortical dementias. J. Neurol. Neurosurg. Psychiatry.

[18] Simonsen A.H., Herukka S.K., Andreasen N., Baldeiras I., Bjerke M., Blennow K., Engelborghs S., Frisoni G.B., Gabryelewicz T., Galluzzi S. (2017). Recommendations for CSF AD biomarkers in the diagnostic evaluation of dementia. Alzheimer’s Dement..

[19] Molinuevo J.L., Blennow K., Dubois B., Engelborghs S., Lewczuk P., Perret-Liaudet A., Teunissen C.E., Parnetti L. (2014). The clinical use of cerebrospinal fluid biomarker testing for Alzheimer’s disease diagnosis: A consensus paper from the Alzheimer’s Biomarkers Standardization Initiative. Alzheimer’s Dement..

[20] del Campo M., Mollenhauer B., Bertolotto A., Engelborghs S., Hampel H., Simonsen A.H., Kapaki E., Kruse N., Le Bastard N., Lehmann S. (2012). Recommendations to standardize preanalytical confounding factors in Alzheimer’s and Parkinson’s disease cerebrospinal fluid biomarkers: An update. Biomark. Med..

[21] Paraskevas G.P., Kasselimis D., Kourtidou E., Constantinides V., Bougea A., Potagas C., Evdokimidis I., Kapaki E. (2017). Cerebrospinal Fluid Biomarkers as a Diagnostic Tool of the Underlying Pathology of Primary Progressive Aphasia. J. Alzheimer’s Dis..

[22] Constantinides V.C., Paraskevas G.P., Boufidou F., Bourbouli M., Stefanis L., Kapaki E. (2021). Cerebrospinal fluid biomarker profiling in corticobasal degeneration: Application of the AT(N) and other classification systems. Parkinsonism Relat. Disord..

[23] Bian H., Van Swieten J.C., Leight S., Massimo L., Wood E., Forman M., Moore P., de Koning I., Clark C.M., Rosso S. (2008). CSF biomarkers in frontotemporal lobar degeneration with known pathology. Neurology.

[24] Mattsson-Carlgren N., Grinberg L.T., Boxer A., Ossenkoppele R., Jonsson M., Seeley W., Ehrenberg A., Spina S., Janelidze S., Rojas-Martinex J. (2022). Cerebrospinal Fluid Biomarkers in Autopsy-Confirmed Alzheimer Disease and Frontotemporal Lobar Degeneration. Neurology.

[25] Forman M.S., Farmer J., Johnson J.K., Clark C.M., Arnold S.E., Coslett H.B., Chatterjee A., Hurtig H.I., Karlawish J.H., Rosen H.J. (2006). Frontotemporal dementia: Clinicopathological correlations. Ann. Neurol..

[26] Kertesz A., McMonagle P., Blair M., Davidson W., Munoz D.G. (2005). The evolution and pathology of frontotemporal dementia. Brain.

[27] Padovani A., Premi E., Pilotto A., Gazzina S., Cosseddu M., Archetti S., Cancelli V., Paghera B., Borroni B. (2013). Overlap between frontotemporal dementia and Alzheimer’s disease: Cerebrospinal fluid pattern and neuroimaging study. J. Alzheimers Dis..

[28] Foiani M.S., Cicognola C., Ermann N., Woollacott IO C., Heller C., Heslegrave A.J., Keshavan A., Paterson R.W., Ye K., Kornhuber J. (2019). Searching for novel cerebrospinal fluid biomarkers of tau pathology in frontotemporal dementia: An elusive quest. J. Neurol. Neurosurg. Psychiatry.

[29] Marelli C., Gutierrez L.A., Menjot de Champfleur N., Charroud C., De Verbizier D., Touchon J., Douillet P., Berr C., Lehmann S., Gabelle A. (2015). Late-onset behavioral variant of frontotemporal lobar degeneration versus Alzheimer’s disease: Interest of cerebrospinal fluid biomarker ratios. Alzheimer’s Dement..

[30] Cousins K.A.Q., Irwin D.J., Wolk D.A., Lee E.B., Shaw L.M.J., Trojanowski J.Q., Da Re F., Gibbons G.S., Grossman M., Phillips J.S. (2020). ATN status in amnestic and non-amnestic Alzheimer’s disease and frontotemporal lobar degeneration. Brain.

[31] Rosso S.M., van Herpen E., Pijnenburg Y.A., Schoonenboom N.S., Scheltens P., Heutink P., van Swieten J.C. (2003). Total tau and phosphorylated tau 181 levels in the cerebrospinal fluid of patients with frontotemporal dementia due to P301L and G272V tau mutations. Arch. Neurol..

[32] Bibl M., Mollenhauer B., Lewczuk P., Esselmann H., Wolf S., Otto M., Kornhuber J., Ruther E., Wiltfang J. (2011). Cerebrospinal fluid tau, p-tau 181 and amyloid-beta38/40/42 in frontotemporal dementias and primary progressive aphasias. Dement. Geriatr. Cogn. Disord..

[33] Constantinides V.C., Paraskevas G.P., Emmanouilidou E., Petropoulou O., Bougea A., Vekrellis K., Evdokimidis I., Stamboulis E., Kapaki E. (2017). CSF biomarkers beta-amyloid, tau proteins and a-synuclein in the differential diagnosis of Parkinson-plus syndromes. J. Neurol. Sci..

[34] Lleo A., Irwin D.J., Illan-Gala I., McMillan C.T., Wolk D.A., Lee E.B., Van Deerlin V.M., Shaw L.M., Trojanowski J.Q., Grossman M. (2018). A 2-Step Cerebrospinal Algorithm for the Selection of Frontotemporal Lobar Degeneration Subtypes. JAMA Neurol..

